# Ethical Leadership and Knowledge Hiding: A Moderated Mediation Model of Relational Social Capital, and Instrumental Thinking

**DOI:** 10.3389/fpsyg.2019.02403

**Published:** 2019-10-25

**Authors:** Muhammad Ibrahim Abdullah, Huang Dechun, Moazzam Ali, Muhammad Usman

**Affiliations:** ^1^Business School of Hohai University, Hohai University, Nanjing, China; ^2^Department of Management Sciences, COMSATS University Islamabad, Lahore Campus, Lahore, Pakistan; ^3^Department of Management Sciences, University of Okara, Okara, Pakistan

**Keywords:** ethical leadership, relational social capital, knowledge hiding, instrumental thinking, moderated mediation

## Abstract

The present study examined the direct and indirect (via relational social capital) relationships between supervisors’ ethical leadership and knowledge hiding. It also tested the moderating role of instrumental thinking in the relationship between supervisors’ ethical leadership and knowledge hiding and the relationship between supervisors’ ethical leadership and relational social capital. Data were collected from 245 employees in different firms spanning different manufacturing and service sectors. The results showed that supervisors’ ethical leadership was negatively related to knowledge hiding, both directly and via relational social capital. The results revealed that instrumental thinking moderated the positive relationship between supervisors’ ethical leadership and relational social capital, such that the relationship was weak when instrumental thinking was high. The results also showed that instrumental thinking moderated both direct and indirect relationships between supervisors’ ethical leadership and knowledge hiding, such that the relationships were weak when instrumental thinking was high. The study carries important practical implications for managers concerned about the destructive consequences of knowledge hiding.

## Introduction

Knowledge hiding – a phenomenon of withholding and concealing knowledge intentionally from others (e.g., peers and managers) who requested it – is a critical issue in the social fabric of a workplace that results in several destructive influences on employees’ and organizations’ productivity and performance (Connelly et al., 2012; Peng, 2013; Connelly and Zweig, 2015; Zhao and Xia, 2019). Knowledge hiding impairs interpersonal relationship dynamics, such as interpersonal trust and the overall quality of relations, and jeopardizes managers’ strategies to enhance employees’ learning and creativity, and stymies managers’ endeavors to help organizations gain a sustained competitive advantage (Connelly et al., 2012; Černe et al., 2014; Connelly and Zweig, 2015; Wang et al., 2019; Zhao and Xia, 2019). Peng (2013) revealed that 46%of respondents from his Chinese sample data acknowledged that they hide knowledge. Connelly et al. (2012) reported that 76% of the respondents from the United States sample hide knowledge requested from them. Babcock (2004) reported that Fortune 500 companies suffer a loss of 31.5 billion US dollars every year due to knowledge hiding, suggesting that knowledge hiding stymies managers’ endeavors to gain competitive advantage. Despite these obvious destructive consequences of knowledge hiding for employees’ work-related behaviors and organizations’ long-term success, how managers can address it remains under-developed both theoretically and empirically (Men et al., 2018). Recent calls (Connelly et al., 2017; Men et al., 2018) rightly highlighted that the literature on the contingencies and antecedents of knowledge hiding is still in its infancy.

In an effort to contribute to this nascent yet growing field of knowledge hiding, the work at hand draws on social learning theory (Bandura, 1977, 1986), social exchange theory (Blau, 1964), and literature on instrumental thinking to propose and test a model that integrates ethical leadership, relational social capital, instrumental thinking, and knowledge hiding. Ethical leadership refers to – “*the demonstration of normatively appropriate conduct through personal actions and interpersonal relationships, and the promotion of such conduct to followers”* (Brown et al., 2005, p. 120). Our interest in studying ethical leadership in relationship with knowledge hiding is inspired mainly by the ethical leadership’s central focus on ethics, the quintessence that differentiates ethical leadership from other leadership styles (e.g., transformational leadership and authentic leadership) (Brown et al., 2005; Brown and Mitchell, 2010; Ng and Feldman, 2015) and its role in shaping employees’ ethical behaviors and discouraging unethical behaviors (Eisenbeiß and Brodbeck, 2014; DeConinck, 2015; Usman and Hameed, 2017; Hoch et al., 2018), including knowledge hiding (Tang et al., 2015; Men et al., 2018). Moreover, although Tang et al. (2015) and Men et al. (2018) have revealed that ethical leadership is negatively related to employees’ knowledge-hiding behaviors, as noted by Men et al. (2018), there is a paucity of research on the mediating mechanisms and boundary conditions of the relationship. Therefore, our primary impetus is to study and bring to the fore the intervening mechanisms and boundary conditions of the relationship between ethical leadership and knowledge hiding.

To build our model, first, the present study draws on social learning theory (Bandura, 1977, 1986) and social exchange theory (Blau, 1964) to introduce relational social capital as a mediating mechanism of the relationship between ethical leadership and knowledge hiding. Relational social capital refers to an employee’s relationships with organizational members that entail high levels of trust in others, affection, reciprocity, care for others, and open interaction (Nahapiet and Ghoshal, 1998; Chang, 2016; Steinmo and Rasmussen, 2018). It is worth noting that as relational social capital refers to employees’ quality of relationship with organizational members (Moran, 2005), it can have different foci/targets, such as managers (leader-member exchange, Graen and Uhl-Bien, 1995) and colleagues. Given our focus on knowledge hiding among peers, we focus on high-quality relationships between peers. Thus, in the present work, relational social capital refers to the quality of relationships of an employee with his/her peers. We consider relational social capital because Connelly et al. (2012) argue that employees’ interpersonal relational dynamics with peers affect their knowledge behaviors and urge scholars to explore further how different patterns of employees’ relationship with peers can influence their knowledge hiding behaviors. Moreover, high-quality relations and the context embedding these relations play an important role in shaping individuals’ perceptions of the self, the context, and others, and can create a sense of congruence between the self and others in the context that can determine individuals’ behaviors (Putnam, 1995; Tsai and Ghoshal, 1998; Uzzi, 1999). Relationships among peers based on affection and a high level of trust are a crucial aspect of the successful execution of their everyday work, and imperative for teamwork, cooperation, the acquisition of unproven and complex knowledge (Strati, 1999; Steinmo and Rasmussen, 2018), and discourage employees’ engagement in deceptive and opportunistic behaviors (Gulati and Gargiulo, 1999; Kale et al., 2000; Yang and Farn, 2009; Connelly et al., 2012). By empirically showing that relational social capital mediates the relationship between ethical leadership and knowledge hiding, the present work advances our understanding of the consequential potential of ethical leadership for shaping high-quality, trust-based relationships among coworkers, as well as the value of such high-quality relationships among coworkers for explaining why ethical leadership is negatively related to knowledge hiding.

Second, the present work suggests that an individual difference factor, instrumental thinking – a preoccupation of an individual with a calculation of means to achieve some narrowly-defined, self-interested ends (Horkheimer, 1974) – act as a boundary condition of the relationship between ethical leadership and knowledge hiding, as well as the relationship between ethical leadership and relational social capital. Instrumental thinking as a moderator carries significant relevance for theory and practice. On the one hand, as employees with high instrumental thinking because of their high economic orientation search apparently for the most cost-effective and direct means to achieve their targets (Horkheimer, 1974; Lee et al., 2015), they can be more effective in helping managers to achieve their economic objectives (Belmi and Pfeffer, 2015; Orehek and Forest, 2016). Therefore, managers often favor those employees who demonstrate high instrumental thinking (Belmi and Pfeffer, 2015). On the other hand, high economic orientation and search for cost-effective means can undermine individuals social orientation that can lead them to engage in deceptive behaviors (Rauthmann, 2012; Watts et al., 2013), such as distorting information and providing incomplete information to the knowledge seeker. Therefore, employees with high instrumental thinking may pose the dilemma – ethics versus productivity – for the leaders.

**FIGURE 1:**
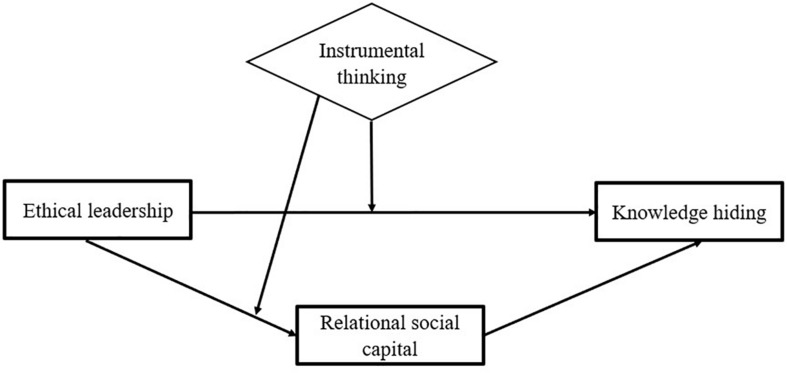
The proposed model.

In terms of interpersonal relationships, people with high instrumental thinking because of their high economic orientation prefer productive relationships over long-term, trust-based relationships (Gruenfeld et al., 2008). Thus, it is important to understand the implications of instrumental thinking for the relationship between ethical leadership and knowledge hiding, and the relationship between ethical leadership and employees’ relational social capital. Together, we present a case of moderated mediation, whereby the strength of the indirect association between ethical leadership and knowledge hiding via relational social capital is contingent upon the levels of instrumental thinking (see Figure 1 for the proposed model). Our focus on the moderating role of instrumental thinking offers a nuanced explanation of why social learning process can have differential effects on different employees, as well as account for differences in their knowledge hiding behaviors and attitudes toward interpersonal relationships.

## Theory and Hypotheses

### Ethical Leadership and Knowledge Hiding

It is worth noting that knowledge hiding is different from other dysfunctional behaviors, such as knowledge hoarding, territoriality, workplace incivility, social undermining, deception, workplace aggression, and a lack of knowledge sharing (see Connelly et al., 2012 for detail). Therefore, knowledge of hiding can have different antecedents and implications for individuals and organizations. In this study, we draw on social learning theory (Bandura, 1977, 1986) and suggest a negative relationship between ethical leadership and employees’ knowledge-hiding behavior. Ethical leaders do “the right thing,” do not compromise their integrity and values in their personal and professional matters, and strive to make balanced and fair decisions that serve the interest of their followers and organizations (Kalshoven et al., 2012; Den Hartog, 2015; Usman and Hameed, 2017). Likewise, ethical leaders demonstrate honesty and altruism through their behaviors and actions (Brown et al., 2005; Eisenbeiß and Brodbeck, 2014). The social learning theory suggests that followers learn such traits as honesty, integrity, and altruism from their leaders. We expect that those followers who demonstrate altruism, honesty, integrity, and, other such features through their behaviors may not engage in deceptive behaviors, such as knowledge hiding.

Tang et al. (2015) and Men et al. (2018) provided empirical evidence of the negative relationship between ethical leadership and knowledge hiding. Men et al. (2018) suggest that an ethical leader’s interactions with his/her followers based on demonstration of openness, care, loyalty, and benevolence encourage them to express their authentic self without fear of destructive repercussions for their career, status, and self-image. Thus, we expect a negative relationship between ethical leadership and knowledge hiding.

As a unit of analysis for ethical leadership, we focus on employees’ immediate supervisors as ethical leaders and their influence on employees’ knowledge-hiding behavior because of the close proximity and frequent interactions of employees with their immediate supervisors (Mayer et al., 2009). Moreover, immediate supervisors play a vital role in rewarding and disciplining employees for their behaviors and performance (Davis and Rothstein, 2006). Therefore, the likelihood of the influence of immediate supervisors on employees behaviors is increased (Davis and Rothstein, 2006; Johnson et al., 2010). Thus, our unit of analysis of ethical leadership is supervisory ethical leadership. The discussion in this subsection informs the following hypothesis.

Hypothesis 1: Supervisors’ ethical leadership is negatively related to knowledge hiding.

### Relational Social Capital as a Mediator

Relational social capital creates a sense of obligation among individuals that intrinsically motivates them to strive for a collective purpose, promote cooperative norms and shapes in them a commitment to reciprocate others’ helping behaviors (Coleman, 1990; Putnam, 1995; Nahapiet and Ghoshal, 1998; Moran, 2005; Yang and Farn, 2009; Berends and Lammers, 2010). Ethical leaders are driven by a broader, collective purpose (Treviño et al., 2003; Brown et al., 2005; Kalshoven et al., 2012). The ethical leader’s focus on a collective purpose increases cooperation among an organization’s members (De Cremer and van Knippenberg, 2002; Treviño et al., 2003; Kalshoven et al., 2012). Moreover, the ethical leader demonstrates a people orientation by demonstrating altruism and consideration for their followers’ welfare through his behaviors and actions (De Cremer and van Knippenberg, 2002; Brown et al., 2005; De Hoogh and Den Hartog, 2008; Moore et al., 2019). Social learning theory (Bandura, 1977, 1986) suggests that the followers will learn altruism (people-orientation and care for others) and integrity from the ethical leader and demonstrate these traits while interacting with their peers. Ethical leaders communicate the value of integrity, altruism, and other such traits to their followers that help the leaders achieve the collective purpose (Brown et al., 2005). That is, the ethical leader inspires his/her followers to demonstrate integrity and altruism through their behaviors. Based on social exchange theory (Blau, 1964), we expect that the followers’ demonstration of altruism, integrity, and other such traits will draw positive reciprocal responses from their peers. Past research (e.g., Fisher and Brown, 1988; Ferrin et al., 2006; De Hoogh and Den Hartog, 2008) suggests that altruistic behaviors (e.g., showing care and concern for others’ interests) create a high level of interpersonal trust, one of the key manifestations of relational social capital. This argument is line with extant research that suggests that ethical leadership’s demonstration of altruism and openness create a work environment that enriches mutual trust among employees, and inspire open interaction, positive feelings and behaviors, such as cooperation and forgiveness (Cameron et al., 2004; Brown et al., 2005; Neubert et al., 2009; Usman et al., 2018b; Moore et al., 2019). As a high level of interpersonal trust, cooperation, open interaction, and altruistic behaviors are the defining characteristics of relational social capital (Yang and Farn, 2009), we expect a positive relationship between supervisors’ ethical leadership and employees’ relational social capital.

Additionally, the relationships based on trust and affection make employees more responsive to each other (Uzzi, 1996). They listen to each other and clarify how different aspects of a practice are interconnected to contribute to the organization’s success (Uzzi, 1996; Hansen, 1999; Moran, 2005; Phelps et al., 2012; Usman et al., 2018a). Relational social capital promotes cooperative norms and a commitment to reciprocate others’ helping behaviors (Coleman, 1990; Putnam, 1995). Moreover, interpersonal relationships based on a high level of trust and affection discourage deceptive and opportunistic behaviors (Gulati and Gargiulo, 1999; Kale et al., 2000; Yang and Farn, 2009). Therefore, we anticipate that employees’ relational social capital is negatively related to knowledge hiding. In sum, we develop the following hypothesis.

*Hypothesis 2: Relational social capital mediates the negative relationship between* supervisors’ *ethical leadership and employees’ knowledge-hiding behavior.*

### Instrumental Thinking as a Moderator

Ethical leaders focus on the achievement of collective goals and take measures to protect the rights of the firm’s stakeholders and demonstrate such a focus through his actions and behaviors (Brown et al., 2005). On the contrary, instrumental thinking is a preoccupation of an individual with a calculation of means to achieve some narrowly-defined, self-interested ends (Horkheimer, 1974), suggesting an incongruence between the guiding principles of ethical leaders and the followers with high instrumental thinking. Prior research suggests that individuals are less receptive of and responsive to the information, actions, and behaviors that are inconsistent with their identities and threaten to impede their self-interested ends (Markus, 1977; Hui et al., 2000). Based on this line of reasoning, we understand that employees with high instrumental thinking may perceive ethical leadership’s emphasis on the achievement of the collective goals as an impediment to their personal objectives. Thus, followers with high instrumental thinking are likely to be less responsive to their leaders’ collective focus, integrity, and altruism. Consequently, employees with high instrumental thinking are less likely to demonstrate altruism, integrity, and other such traits through their behaviors while interacting with their peers. Therefore, we argue that high instrumental thinking can impede the positive influence of ethical leadership on relational social capital.

Second, the literature on instrumental thinking suggests that interpersonal relationships based on instrumental thinking are deprived of warmth and affection, as the identification of a need initiates a relationship between two parties and the fulfillment of the need terminates it (Horkheimer, 1974). Instrumental thinking manifests the features of searching the most cost-effective and direct means to realize a desired objective (Horkheimer, 1974; Labroo and Kim, 2009; Lee et al., 2015). The parties that engage in an instrumental relation are bargainers and thus, they are not seeking to cultivate affection-based durable relations (Horkheimer, 1974). Instead, they enter the relationships instrumentally (treating people as objects – as an effective means to advance their personal interests) to seek satisfaction of some personal need (Lee et al., 2015). Oakeshott (1991) uses the term ‘enterprise association’ for such relationships, negotiated and formed between self-interested bargainers founded on harmonization between the interests of the bargainers. Thus, it is expected that employees with high instrumental thinking can be less interested in developing and maintaining high-quality, trust-based relationships with their peers. In sum, the following hypothesis is developed.

*Hypothesis 3: Instrumental thinking moderates the positive relationship between* supervisors’ *ethical leadership and relational social capital, such that the relationship is weak when instrumental thinking is high.*

Instrumental attitude’s manifestations are common in contemporary work settings and organizations’ relationships with their employees and customers (Tenbrunsel and Messick, 1999; Orehek and Forest, 2016). In work settings, individuals are inclined to think strategically and instrumentally. For instance, top managements’ choices are based on strategic thinking, and those choices are in favor of those people whom they perceive can be helpful and instrumental as a means to achieve the desired ends (Tenbrunsel and Messick, 1999; Belmi and Pfeffer, 2015; Belmi and Laurin, 2016; Orehek and Forest, 2016). Several studies have revealed that individuals striving to achieve self-interested ends tend to engage in lying, corruption, and other unethical and deceptive behaviors (Steidlmeier, 1999; Sendjaya and Cooper, 2011; Rauthmann, 2012; Watts et al., 2013), such as withholding the requested knowledge (Gkorezis and Bellou, 2016). Self-interested individuals prioritize the self against the collective interest (Rus et al., 2012; Wisse and Rus, 2012) and withhold information from others in the group and the organization, as they perceive information sharing as a threat to the self (Gkorezis and Bellou, 2016). Building from this line of reasoning, it is inferred that employees with high self-interest (instrumental thinking) may consider the provision of the requested knowledge as a more serious threat to the self that those with low self-interest (instrumental thinking). Therefore, it can be expected that employees with high instrumental thinking are more likely to hide knowledge than those with low instrumental thinking.

Additionally, according to Marx (1844/1964), individuals with primacy to personal riches and personal motives are less concerned about the qualities (e.g., altruism, honesty, and the ethicality of the processes) that define humanity. To serve their personal interests, such as to maximize their monetary benefits, self-centered people may engage in unethical decision-making and unethical behaviors (Rijsenbilt and Commandeur, 2013), suggesting a perceived incongruence between the guiding principles of ethical leadership and employees preoccupied with instrumental thinking. Such an incongruence can be more pronounced between ethical leadership and employees with high instrumental thinking. Past research indicates that a mismatch between the interests of the two parties is perceived as an obstruction to the achievement of their respective goals and can also be construed as a threat to their respective identities (Markus, 1977; Hui et al., 2000; Zhang and Bloemer, 2011). Consequently, employees with high instrumental thinking are likely to be less responsive to ethical leaders’ characteristics, such as integrity, honesty, altruism, and shared values. Together, we expect that high instrumental thinking may mitigate the influence of ethical leadership on knowledge hiding.

*Hypothesis 4: Instrumental thinking moderates the negative relationship between* supervisors’ *ethical leadership and knowledge hiding, such that the relationship is weak when instrumental thinking is high.*

As alluded above (hypothesis 4), high instrumental thinking can mitigate the positive influence of ethical leadership on employees’ relational social capital, indicating that the mediating role of relational social capital in the negative relationship between ethical leadership and knowledge hiding is contingent upon the level of instrumental thinking. From a statistical point of view, we present a moderated mediation case (Hayes, 2013). In other words, the amount to which relational social capital (the mediator) translates the influence of ethical leadership (the predictor) on knowledge hiding (the outcome variable) may depend on the level of instrumental thinking (the moderator). Thus, the following hypothesis was developed.

*Hypothesis 5: The indirect relationship between* supervisors’ *ethical leadership and knowledge hiding via relational social capital is moderated by instrumental thinking, such that the indirect relationship is weaker for those with high instrumental thinking than for those with low instrumental thinking.*

## Research Method

### Data Collection and Analysis

Time-lagged survey data (three rounds, 2 months apart) were collected from 245 alumni of a large public sector university in Pakistan. The participants were full-time employees in various manufacturing and service sectors, including banking, health, insurance, information technology, restaurant, cement, textile, and ceramics. Employees’ knowledge-hiding behaviors and their perceptions of ethical leadership might vary across professions, organizations, and industries. Therefore, to capture maximum variance in knowledge hiding and ethical leadership, as well as enhance the generalizability of our findings, data were collected from a heterogeneous sample. Previous studies (e.g., Kreiner, 2006; Hirschi, 2012; Abbas et al., 2014) suggest that a heterogeneous sample helps capture maximum variance in the important constructs of a study and enhances the generalizability of findings.

Data were collected in three rounds to avoid common method bias (Podsakoff et al., 2003). A 2-month lag time is considered long enough to reduce the possibility that the respondents would recall and relate previous responses with the current responses (Peng, 2013). Moreover, Harman’s single-factor test was used to diagnose common method bias (Hu and Bentler, 1999). To do so, we constrained the items of all the variables of our study into one factor, which explained 29.23% of the total variance, which was well below the cut-off point of 50% (Hu and Bentler, 1999). As the respondents held either a master’s degree or had completed an undergraduate degree, English-language questionnaire was used for data collection. The survey questionnaire was pre-tested with five academicians and 10 respondents.

We randomly chose 300 alumni from the list of 3142 alumni, who confirmed their participation in a dinner hosted by the university. However, 15 of those randomly chosen alumni were entrepreneurs and were excluded from the list of the potential respondents, as the objective of the study was to understand employees’ knowledge hiding behaviors and how these behaviors can be discouraged. Thus, we initially contacted 285 alumni, who gave written informed consent to participate in all the three rounds of data collection. They were provided with an information sheet, containing the information about the purpose of the study, and the promise of confidentiality.

The first round of data collection was completed by 282 (almost 99% response rate) respondents on the same day after dinner. In the first round, data about ethical leadership, instrumental thinking, age, gender, education, and work experience were collected. The data about relational social capital were collected in the second round; while data about knowledge hiding were collected in the third round. Data in the second round were collected by mailing the questionnaires and pre-paid return envelops. We received 265 (93% response rate) and 254 (96% response rate) responses in the second and the third rounds, respectively. Nine responses with missing data were eliminated. In total, data from 245 employees were used for testing our hypotheses. For all 245 responses, one employee rated one supervisor for ethical leadership. Responses were matched using a unique code.

The final sample included 117 (47.76%) male and 128 (52.24%) female respondents. The average age and experience (the number of years that a person has been employed, Khan et al., 2019) of the respondents were 36.42 and 7.22 years, respectively. In terms of education, 49% had undergraduate degrees, and 51% held master degrees or above. The statistical package for the social sciences (SPSS) 24.0, AMOS 24.0, and Hayes’ PROCESS macro were used to test the hypothesized relationships.

### Measures and Variables

Unless otherwise stated, the items that measured all of the variables in this study were rated on five points – from 1 (strongly disagree) to 5 (strongly agree). All the items were coded such that high scores equated with the higher levels of the constructs.

Supervisors’ ethical leadership was measured using a 10-item scale developed and validated by Brown et al. (2005). *“My supervisor can be trusted,”* was a sample item. Relational social capital was measured by adapting a six-item scale developed and validated by Chang (2016). *“My relationships with my colleagues are characterized by mutual friendship,”* was a sample item. Knowledge hiding was measured using a 12-item scale developed and validated by Connelly et al. (2012). *“I agreed to help him/her but instead gave him/her information different from what s/he wanted,”* was a sample item. As we intended to examine the interrelations between ethical leadership, relational social capital, and the overall level of knowledge hiding, we followed prior research (Černe et al., 2014; Tang et al., 2015; Men et al., 2018) and used an overall measure of knowledge hiding. The fit indices were as follow: χ^2^(50) = 127.82, χ2/df = 2.56, GFI = 0.92, IFI = 0.95, TLI = 0.94, CFI = 0.95, and RMSEA = 0.08. Thus, we averaged the responses to all the 12 items to form an overall measure of the variable. Instrumental thinking was measured by adapting a three-item scale developed and validated by Belmi and Pfeffer (2018). *“I develop relationships with people, including my colleagues by mainly considering how beneficial they might be for me,”* was a sample item.

### Control Variables

Differences in age, gender, work experience, and education can affect ethical judgment, decisions, and behavior (Gilligan, 1982; Marcus and Schuler, 2004) and thus, can affect relational social capital (Yang and Farn, 2009; Chang, 2016) and knowledge hiding (Peng, 2013). However, as work experience, age, education, and gender did not show significant correlations with our outcome variable and the mediator (Table 1), we followed Becker (2005) to present our results without controls.

**TABLE 1:** Means and correlations.

**Construct**	**Means**	**SD**	**1**	**2**	**3**	**4**	**5**	**6**	**7**
(1) Ethical leadership	3.46	0.89							
(2) Relational social capital	3.37	1.04	0.35^∗∗^						
(3) Knowledge hiding	2.35	0.84	–0.27^∗∗^	–0.32^∗∗^					
(4) Instrumental thinking	3.60	1.08	0.11	0.28^∗∗^	–0.19^∗∗^				
(5) Age	36.42	7.68	0.02	0.09	–0.01	0.14^∗^			
(6) Gender	1.52	0.50	–0.06	0.02	0.07	0.01	–0.06		
(7) Education	1.51	0.50	−0.13^∗^	–0.01	0.05	–0.04	–0.01	0.18^∗∗^	
(8) Experience	7.22	5.33	0.03	0.03	0.03	0.14^∗^	0.83^∗∗^	–0.07	0.04

**n* = 245. ^∗^*p* < 0.05. ^∗∗^*p* < 0.01 level (two-tailed). SD = standard deviation. Gender: 1 = male, 2 = female. Education: 1 = undergraduate university degree; 2 = master’s degree or above.*

## Results

### Multicollinearity Test

Data were tested to see if the assumption of collinearity is satisfied. Since the highest variance inflation factor (VIF) value for the variables was 1.21, and the tolerance values ranged between 0.82 and 0.96, the multicollinearity was not a concern (Hair et al., 2010).

### Means and Correlations

Means and correlations for the variables of the study are presented in Table 1.

### Measurement Model

Confirmatory factor analysis was used to evaluate the measurement model, which consisted of supervisors’ ethical leadership, relational social capital, knowledge hiding, and instrumental thinking. The fit indices, χ^2^ (425) = 987.35, χ2/df = 2.32, IFI = 0.90, TLI = 0.90, CFI = 0.90, and RMSEA (IC-90%) = 0.067–0.078, show that the measurement model has an acceptable fit with the data. The scale items and factor loadings are presented in Table 2. Table 3 presents the values of Cronbach’s alpha (α), composite reliability (CR), and average variance extracted (AVE) of all the variables. The scales showed satisfactory levels of internal consistency (α > 0.70). Maximum shared variance (MSV), average shared variance (ASV), and the square root values of AVE are also presented in Table 3. The square root values of AVEs for all the variables were greater than their inter-construct correlations, and ASV and MSV < AVE. Thus, the scales also demonstrated satisfactory levels of discriminant validity and convergent validity.

**TABLE 2:** Items and factor loadings.

**Items**	**Loadings**
**Supervisors’ ethical leadership**	
My supervisor listens to what employees have to say	0.68
My supervisor disciplines employees who violate ethical standards	0.73
My supervisor conducts his/her personal life in an ethical manner	0.91
My supervisor has the best interests of employees in mind	0.88
My supervisor makes fair and balanced decisions	0.77
My supervisor can be trusted	0.68
My supervisor discusses business ethics or values with employees	0.76
My supervisor sets an example of how to do things the right way in terms of ethics	0.78
My supervisor defines success not just by results but also the way that they are obtained	0.66
When making decisions, my supervisor asks “what is the right thing to do?”	0.71
**Relational social capital**	
I believe I can rely on people in my organization without any fear that they will take advantage of me, even if the opportunity arose	0.78
People in my organization will always keep the promises they make to me	0.94
My relationships with my colleagues are characterized by mutual friendship	0.94
My relationships with my colleagues are characterized by high levels of reciprocity	0.71
I believe that people in my organization approach his or her job with professionalism and dedication	0.66
Given track record, I saw no reason to doubt competence and preparation of people in my organization	0.74
**Knowledge hiding**	
I agreed to help him/her but never really intended to	0.74
I agreed to help him/her but instead gave him/her information different from what s/he wanted	0.81
I told him/her that I would help him/her out later but stalled as much as possible	0.85
I offered him/her some other information instead of what he/she really wanted	0.68
I pretended that I did not know the information	0.70
I said that I did not know, even though I did	0.79
I pretended I did not know what s/he was talking about	0.77
I said that I was not very knowledgeable about the topic	0.80
I explained that I would like to tell him/her, but was not supposed to	0.83
I explained that the information is confidential and only available to people on a particular project	0.78
I told him/her that my boss would not let anyone share this knowledge	0.80
I said that I would not answer his/her questions	0.72
**Instrumental thinking**	
I develop relationships with people, including my colleagues by mainly considering how beneficial they would be for me	0.73
I develop relationships with people, including my colleagues by mainly considering how useful they might be for me	0.82
I develop relationships with people, including my colleagues by mainly considering how valuable they might be for me	0.75

**TABLE 3:** Reliability and convergent and discriminant validities.

**Construct**	**1**	**2**	**3**	**4**	**α**	**AVE**	**MSV**	**ASV**
(1) Ethical leadership	**0.76**				0.93	0.58	0.14	0.10
(2) Relational social capital	0.38	**0.80**			0.91	0.64	0.15	0.13
(3) Knowledge hiding	−0.36	−0.39	**0.77**		0.90	0.60	0.15	0.11
(4) Instrumental thinking	0.11	0.33	−0.25	**0.77**	0.81	0.59	0.11	0.06

**n* = 245. MSV = Maximum shared variance. ASV = Average shared variance. Bolded values on the diagonals of columns 2 to 5 are the square root values of AVE.*

### Mediation Results

To test hypotheses 1 and 2, we used Hayes’ PROCESS 4 (5000 bootstrapping was specified). Hypothesis 1, regarding the negative relationship between supervisors’ ethical leadership and knowledge hiding, was supported (*B* = −0.17, 95% confidence interval did not include zero, −0.28 to −0.05). The results (Table 4) also showed that there was a significant negative indirect relationship between supervisors’ ethical leadership and knowledge hiding (*B* = −0.08, 95% confidence interval did not include zero, −0.15 to −0.03). Thus, hypothesis 2 was also supported.

**TABLE 4:** Mediation results – relational social capital mediates the relationship between ethical leadership and knowledge hiding (PROCESS model 4, 95% CI).

	**Bootstrapped CI 95%**						
	
	***B***	***SE***	***t***	***P***	**LL**	**UL**	**R^2^**
**Model 1:** mediator variable model	Outcome: relational social capital						
Ethical leadership	0.40	0.07	5.76	0.00	0.26	0.54	0.12
**Model 2:** outcome variable model	Outcome: Knowledge hiding						
Relational social capital	−0.20	0.05	−3.98	0.00	−0.31	−0.10	0.13
Ethical leadership	−0.17	0.06	−2.78	0.00	−0.28	−0.05	
**Bootstrapping results for the indirect effect**							
The indirect effect of ethical leadership on knowledge hiding via relational social capital	−0.08	0.03			−0.15	−0.03	

**N* = 245, *B* = Unstandardized regression coefficients. Bootstrap sample size = 5000. LL = Lower limit. CI = Confidence interval. UL = Upper limit.*

### Moderation Results

To test the moderating role of instrumental thinking in the relationship between supervisors’ ethical leadership and relational social capital (hypothesis 3) and the relationship between supervisors’ ethical leadership and knowledge hiding (hypothesis 4), as well as moderated mediation where instrumental thinking moderates the indirect relationship (via relational social capital) between supervisors’ ethical leadership and knowledge hiding (hypothesis 5), Hayes’ PROCESS model 8 was used (Table 5). By using Hayes’ PROCESS model 8, the effects of a moderator on the direct and indirect relationships between the independent and the outcome variable and the direct relationship between the independent variable and the mediator can be simultaneously tested. As this study aimed to test the moderating effects of instrumental thinking on the direct relationship between supervisors’ ethical leadership and relational social capital (hypothesis 3), the relationship between supervisors’ ethical leadership and knowledge hiding (hypothesis 4), and the indirect (via relational social capital) relationship between supervisors’ ethical leadership and knowledge hiding (hypothesis 4), the model 8 was used that tested all these hypotheses simultaneously.

**TABLE 5:** Moderated mediation analysis – instrumental as moderates the direct and indirect relationship between ethical leadership and knowledge hiding (PROCESS model 8, 95% CI).

	**Bootstrapped CI 95%**						
	
	**B**	**SE**	**T**	**P**	**LL**	**UL**	**R^2^**
**Model 1:** mediator variable model	Outcome: relational social capital						
Ethical leadership	0.93	0.23	4.10	0.00	0.49	1.38	0.20
Instrumental thinking	0.77	0.21	3.58	0.00	0.34	1.19	
Ethical leadership × Instrumental thinking	–0.15	0.06	–2.58	0.01	–0.27	–0.04	
**The conditional direct effect of ethical leadership on relational social capital**							
Instrumental thinking (−1 SD)	0.58	0.11	5.51	0.00	0.37	0.74	
Instrumental thinking (+1 SD)	0.23	0.09	2.61	0.01	0.06	0.40	
**Model 2:** outcome variable model	Outcome: knowledge hiding						
Ethical leadership	–0.74	0.19	–3.84	0.00	–1.12	–0.36	0.17
Relational social capital	–0.15	0.05	–2.91	0.00	–0.26	–0.05	
Instrumental thinking	–0.63	0.18	–3.49	0.00	–0.98	–0.27	
Ethical leadership × Instrumental thinking	0.15	0.05	3.13	0.00	0.06	0.25	
**The conditional direct effect of ethical leadership on knowledge hiding**							
Instrumental thinking (−1 SD)	–0.36	0.09	–4.20	0.00	–0.52	–0.21	
Instrumental thinking (+1 SD)	–0.03	0.07	–0.39	0.70	–0.17	0.11	
**Bootstrapping results for the indirect effect (via relational social capital)**							
Index of moderated mediation	0.023	0.01			0.001	0.06	
**The conditional indirect effect of ethical leadership on knowledge hiding (via relational capital)**							
Instrumental thinking (−1 SD)	–0.09	0.04			–0.17	–0.02	
Instrumental thinking (+1 SD)	–0.03	0.02			–0.09	0.001	

**N* = 245. B = Unstandardized regression coefficients. Bootstrap sample size = 5000. LL = Lower limit. CI = Confidence interval. UL = Upper limit.*

**FIGURE 2:**
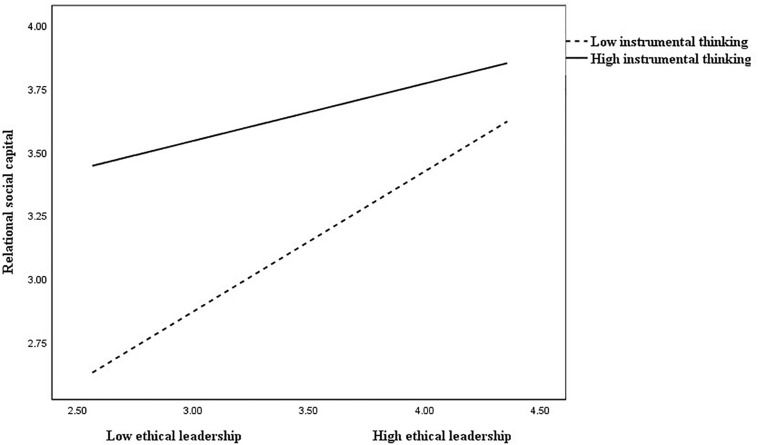
The moderating effect of instrumental thinking on the relationship between ethical leadership and relational social capital.

The results revealed that the effect of the interaction term between supervisors’ ethical leadership and instrumental thinking on relational social capital was significant (*B* = −0.15, *p* < 0.05), suggesting that instrumental thinking moderated the positive relationship between supervisors’ ethical leadership and relational social capital. These interactions were plotted at + 1/−1 SD from the mean of instrumental thinking (Figure 2). Simple slope test was conducted to examine the strength of the relationship between supervisors’ ethical leadership and relational social capital at high and low levels of instrumental thinking. The results showed that the relationship was strong (*B* = 0.58, *p* < 0.001) when instrumental thinking was low; while the relationship was weak (*B* = 0.23, *p* < 0.01) when instrumental thinking was high. Thus, hypothesis 3 was supported.

**FIGURE 3:**
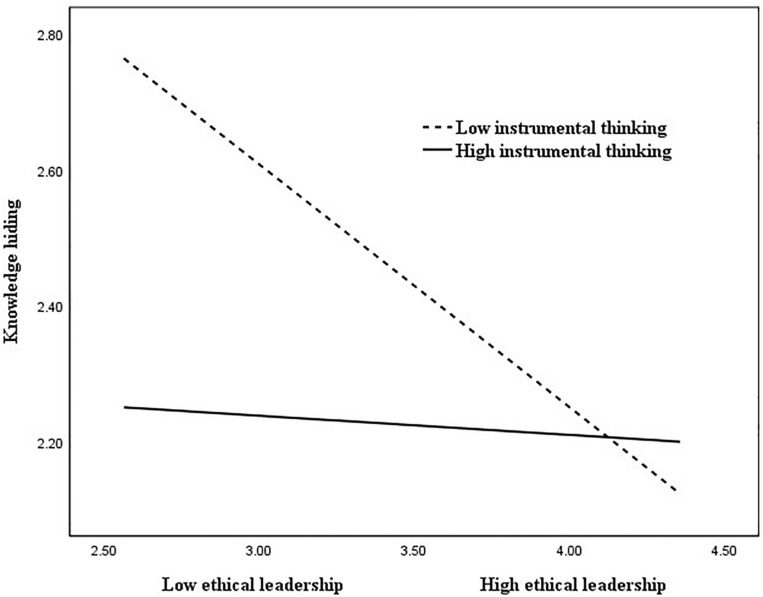
The moderating effect of instrumental thinking on the relationship between ethical leadership and knowledge hiding.

It was also found that the effect of the interaction term between supervisors’ ethical leadership and instrumental thinking on knowledge hiding was significant (*B* = 0.15, *p* < 0.01), suggesting that instrumental thinking moderated the negative relationship between supervisors’ ethical leadership and knowledge hiding. These interactions were plotted at + 1/−1 SD from the mean of instrumental thinking (Figure 3). Simple slope test was conducted to examine the strength of the relationship between supervisors’ ethical leadership and knowledge hiding at high and low levels of instrumental thinking. The results show that the relationship was significant (*B* = −0.36, *p* < 0.001) when instrumental thinking was low; while the relationship was non-significant (*B* = −0.03, ns) when instrumental thinking was high. Thus, hypothesis 4 was supported.

Finally, the results revealed that instrumental thinking moderated the indirect relationship (via relational social capital) between supervisors’ ethical leadership and knowledge hiding [bootstrap estimate = 0.023, bias-corrected CI (0.001, 0.06)]. As shown in Table 5, at low instrumental thinking (−1 SD), the negative indirect relationship between supervisors’ ethical leadership and knowledge hiding was significant. However, at high instrumental thinking (−1 SD), the negative indirect relationship between supervisors’ ethical leadership and knowledge hiding was non-significant (Table 5). Thus, hypothesis 5 was supported.

## Discussion

Given the detrimental effects of employees’ knowledge-hiding behaviors on employees and organizations (Connelly and Zweig, 2015; Bogilović et al., 2017) and the scarcity of research on how to counter such behaviors, the present work based on a three-wave study show that supervisory ethical leadership was negatively related to knowledge hiding. We also found that relational social capital mediated the negative relationship of supervisory ethical leadership and knowledge hiding. Importantly, the results revealed that instrumental thinking moderated the negative relationship between ethical leadership and knowledge hiding, such that the relationship is weaker when instrumental thinking is high. Likewise, the results revealed that instrumental thinking moderated the positive relationship between ethical leadership and relational social capital, such that the relationship was weak when instrumental thinking was high. Finally, we found that instrumental thinking moderated the indirect (via relational social capital) relationship between ethical leadership and knowledge hiding, such that the indirect relationship was weak when instrumental thinking was high.

The present work enhances our understanding of why and when ethical leadership is negatively related to knowledge hiding. Specifically, the study established relational social capital as an important intervening mechanism that explains why supervisors’ ethical leadership is negatively related to knowledge hiding. In line with social learning theory (Bandura, 1977, 1986) and social exchange theory, our findings indicate that ethical leaders’ demonstration of altruism and openness enrich employees’ relational social capital in the form of mutual trust and cooperation that, in turn, discourage followers’ engagement in knowledge-hiding behaviors. As past research has usually focused on close ties between ethical leaders and their followers (Brown and Treviño, 2006; Walumbwa and Schaubroeck, 2009) to explain the relationship between ethical leadership and employees’ work-related attitudes and behaviors, our findings that ethical leaders can discourage employees’ knowledge-hiding behaviors by improving employees’ relational social capital provides a different vantage point to look at the relationship between ethical leadership and employees’ work-related outcomes. Moreover, Tang et al. (2015) and Men et al. (2018) have studied intra-personal/intra-psychic processes, such as psychological safety and psychological empowerment as mediators of the relationship between ethical leadership and knowledge hiding, respectively. The present work departs markedly from these studies by focusing on interpersonal relational dynamics to explain why ethical leadership is negatively related to knowledge hiding.

Additionally, the present study revealed instrumental thinking as a boundary condition of the relationship between supervisors’ ethical leadership and relational social capital, as well as the direct and indirect (via relationship social capital) relationship between ethical leadership and knowledge hiding. Our findings suggest that the incongruence between the interests of an ethical leader and employees with high instrumental thinking can affect the extent to which the followers imitate their leader’s ethical behaviors and thus can explain the differences in the followers’ attitudes toward interpersonal relationships and their knowledge-hiding behaviors. The incongruence of perspectives between an ethical leader and employees with high instrumental thinking can be more profound. Such an incongruence can reduce the effectiveness of the ethical leader’s role modeling role significantly, mitigate the negative influence of supervisors’ ethical leadership on employees’ relational social capital, and dampen the strength of the direct and indirect (vial relational social capital) between ethical leadership and knowledge hiding. In sum, we suggest that depending on the levels of instrumental thinking, individuals may show different levels of responsiveness to their leaders’ behaviors and thus, varying levels of supervisors’ ethical leadership’s influence on employees’ relational social capital and their knowledge-hiding behaviors can be observed. In doing so, we offered a nuanced explanation of why social learning process can have differential effects on different employees and why, as compared with others, some employees can be less responsive to their leaders’ ethical behaviors.

### Theoretical Contributions

Our study contributes to the literature in several ways. First, by revealing relational social capital as a mediator of the relationship between supervisors’ ethical leadership and knowledge hiding, we contributed to the literature on the links between supervisors’ ethical leadership and knowledge hiding (Tang et al., 2015; Men et al., 2018). By doing so, we advanced the scope of role modeling role of supervisors’ ethical leadership by indicating that ethical leaders’ demonstration of integrity, honesty, and altruism through their behaviors can improve their followers’ relational social capital. Given the scarcity of research (Tang et al., 2015; Men et al., 2018) on the mediating mechanisms of supervisors’ ethical leadership and knowledge hiding, this contribution is timely and relevant.

Second, contemporary literature provides evidence about the positive effects of relational social capital on several employees’ work-related behaviors, attitudes, and performance outcomes, such as knowledge acquisition, knowledge sharing, individual and organizational learning, and job performance (Gulati and Gargiulo, 1999; Kale et al., 2000; Yang and Farn, 2009). However, the mediating role of relational social capital as a mediator of the relationship between ethical leadership and knowledge hiding has not been studied. By empirically showing that relational social capital mediates the negative relationship between ethical leadership and knowledge hiding, we advanced the nomological networks of antecedents and outcomes of relational social capital and also presented relational social capital as a potential means for deterring knowledge hiding. In doing so, we responded to the recent calls (e.g., Connelly et al., 2012; Wang et al., 2019; Zhao and Xia, 2019) to further explore the influence of employees’ interpersonal relational dynamics on their knowledge-hiding behaviors.

Third, there is a paucity of research on the boundary conditions of the relationship between ethical leadership and knowledge hiding. Specifically, to date, no study has provided empirical evidence of the individual differences as boundary conditions of the direct and indirect (via relational social capital) relationships between ethical leadership and knowledge hiding. We contributed to the literature on the links between ethical leadership and knowledge hiding (Tang et al., 2015; Men et al., 2018) by theorizing and providing evidence that instrumental thinking, an individual difference factor, acts as a boundary condition of the relationship between ethical leadership and employees’ relational social capital. Likewise, the work at hand provided evidence that instrumental thinking moderates the direct and indirect relationships between ethical leadership and knowledge hiding, In doing so, the present work responded to the calls for further research unveil the interaction effects of ethical leadership and individual differences on the relationship between ethical leadership and employees’ work-related attitudes and behaviors (Kacmar et al., 2013; Men et al., 2018; Yang and Wei, 2018; Moore et al., 2019), as well as the calls for further investigation into the contingencies of the relationship between ethical leadership and knowledge hiding behaviors (Tang et al., 2015; Men et al., 2018).

Finally, by revealing that instrumental thinking as the boundary condition of the relationship between supervisors’ ethical leadership and knowledge hiding, we provided important insight into the role of instrumental thinking in the organizational context. Several scholars have indicated that instrumental thinking is linked with several dysfunctional and unethical behaviors, such as deception, corruption, bending, cheating, and other unethical behaviors (Rauthmann, 2012; Rijsenbilt and Commandeur, 2013; Watts et al., 2013). However, to date, to the best of our knowledge, the role of instrumental thinking as a moderator of the relationship between ethical leadership and employees’ knowledge-hiding behaviors is not yet known. Thus, our study is important, because it suggests the researchers and practitioners to pay attention to individuals who are high on instrumental thinking to appreciate the intricacies enmeshed in the leader-follower interaction while attempting to address employees’ knowledge-hiding behaviors.

### Practical Implications

Our study provides valuable insight into how managers can discourage employees’ knowledge-hiding behaviors. It is suggested that supervisors can play an important role in discouraging followers’ knowledge-hiding behaviors by facilitating the formation of followers’ relational social capital. Supervisors can do so by demonstrating honesty, integrity, altruism, and other such traits through their behaviors and actions. The present study also suggests that top leadership needs to encourage supervisors to demonstrate honesty, integrity, altruism through their behaviors and actions to inspire their followers to imitate such behaviors that would help the followers develop trust-based relationships with their colleagues and enhance cooperation among the followers. The trust-based relationships among the followers would encourage them to extend selfless care for their colleagues’ personal and professional information and knowledge needs rather than hiding knowledge from them.

However, we insist that managers should focus on understanding the individual differences that can mitigate the effects of their ethical behaviors on employees. This study informs managers about employees’ knowledge-hiding behaviors that can emerge as a result of high instrumental thinking and brings to the fore why some employees, unlike others, see value in high-quality relationships with coworkers. Depending on the levels of instrumental thinking, individuals may show different levels of responsiveness to their leaders’ behaviors, and thus we may observe varying levels of ethical leadership’s influence on employees’ relational social capital and their knowledge-hiding behaviors. Understanding such roles of instrumental thinking are particularly relevant when employees with high instrumental thinking can be seen as a more effective means of achieving economic objectives. Understanding the attitudes and behaviors of employees with, both high and low instrumental thinking can help managers address the dilemma of ‘ethics versus productivity.’ Specifically, managers need to pay attention to employees with high instrumental thinking, because such employees may not give much weight to ethical leaders’ pro-social characteristics as honesty and altruism that are important for developing employees’ trust-based relationships with their colleagues and deterring their knowledge-hiding behaviors.

Although leaders can pay customized attention to employees with high instrumental thinking to discourage such thinking by advocating the importance of pro-social values, such as honesty and altruism, we suggest that hiring decisions should go beyond from consideration for individuals’ competence to understand their levels of instrumental thinking. According to Lee et al. (2015), in organizational contexts, monetary rewards, personal development, and personal career growth are important determinants of a person’s instrumental thinking. Moreover, individuals high on instrumental thinking act forcefully and assertively to send competence signals to those who observe them (Anderson and Kilduff, 2009). We suggest that managers responsible for hiring decisions should understand such signals as the manifestations of high instrumental thinking and avoid hiring people based on competence alone, as it could prove a short-sighted strategy. For this purpose, managers should be trained in creating a balance between employees’ social and economic orientations.

### Limitations and Future Research

This study has certain limitations that should be considered when interpreting the results of the present study. First, the findings are based on time-lagged survey data collected from the same source. Although the time-lagged data reduces common method bias (Podsakoff et al., 2003), our research may restrict causal inferences. We encourage the use of experimental longitudinal designs to draw causal inferences. Moreover, following previous studies on knowledge hiding (Connelly et al., 2012; Černe et al., 2014; Men et al., 2018), we relied on self-reports of knowledge hiding. Peers’ reports on employees’ knowledge-hiding behaviors can offer valuable insight into the studied relationships. Proceeding further, regarding ethical leadership, we focused on supervisors’ ethical behaviors; future studies could collect data from top management and examine its trickle-down effects on knowledge-hiding behaviors of different management layers and employees. Although the heterogeneous samples enhance the generalizability of the findings (Hirschi, 2012; Abbas et al., 2014), all the respondents of our study were university-graduated and white-collar employees. Therefore, it is important to investigate the proposed model by collecting data from blue-collar workers with different educational levels.

Likewise, knowledge hiding can exist in every organization, as well as managers in different organizations can demonstrate some common traits of ethical leadership, and therefore, our findings can be generalized to different contexts and organizations. However, our findings are based on a relatively small sample that belonged to companies operating in a collectivist culture. The perceptions of ethical leadership and the level of knowledge hiding may vary across cultures and contexts (Černe et al., 2014). Therefore, further investigations of our proposed model in different contexts and cultures based on large samples can facilitate better generalization of our findings.

Additionally, the dimensions of ethical leadership – trustworthiness, honesty, care for employees’ professional and personal interests, integrity, fairness, and justice (Brown et al., 2005) – overlap with an organizational virtuousness context, which encourages cooperation, forgiveness and trust (Cameron et al., 2004) and can discourage employees’ engagement in destructive behaviors such as knowledge-hiding behaviors. Therefore, organizational virtuousness context can be a potential mediating mechanism between ethical leadership and employees’ knowledge-hiding behaviors and can provide an avenue for future research. Likewise, other positive leadership styles, such as spiritual leadership (Fry, 2003) can create conditions based on altruistic love and positive emotions that can impede knowledge hiding and therefore, offer an interesting future research agenda.

Furthermore, the loss of authority, power and job insecurity are among the factors that contribute to knowledge-hiding behaviors (Connelly et al., 2012), suggesting that job security can be among the factors that can deter employees’ knowledge-hiding behaviors. Future studies could examine the interrelations between ethical leadership, job security, and knowledge-hiding behaviors. Finally, individual difference factors, such as meaningful work, can also act as boundary conditions of the relationship between ethical leadership and knowledge hiding. For instance, employees with high perceptions of meaningful work can demonstrate more social orientation and go beyond the norms to fulfill their colleagues’ knowledge needs. Therefore, it can be expected that such employees may not involve in information distortion and other such behaviors. Moreover, employees with high perceptions of meaningful work can be more responsive to ethical leaders’ ethical and pro-social behaviors and thus, can inflate the influence of ethical leadership on relational social capital and knowledge hiding.

## Data Availability Statement

The datasets generated for this study are available on request to the corresponding author.

## Ethics Statement

This study involving human participants was reviewed and approved by the Ethics Committee of the Department of Management Sciences, COMSATS University Islamabad, Lahore Campus, Lahore, Pakistan. The participants provided their written informed consent to participate in this study.

## Author Contributions

MIA, HD, MU, and MA: definition of research objectives, models, hypotheses, data analysis plan, principal article writing, article revision and proofreading, and final approval. MA and MU: the provision of materials (i.e., questionnaires) and data collection. MIA, MA, and MU: data analysis.

## Conflict of Interest

The authors declare that the research was conducted in the absence of any commercial or financial relationships that could be construed as a potential conflict of interest.
